# Inventory study of an early pandemic COVID-19 cohort in South-Eastern Sweden, focusing on neurological manifestations

**DOI:** 10.1371/journal.pone.0280376

**Published:** 2023-01-13

**Authors:** Doryaneh Ahmadpour, Anna Kristoffersson, Mats Fredrikson, Yumin Huang-Link, Anne Eriksson, Ellen Iacobaeus, Anne-Marie Landtblom, Sara Haghighi

**Affiliations:** 1 Department of Biology and Biological Engineering, Chalmers University of Technology, Gothenburg, Sweden; 2 Department of Medical Specialists, Institute of Neurology, Motala Hospital, Motala, Sweden; 3 Forum Östergötland, Linköping University, Linköping, Sweden; 4 Department of Neurology, Linköping University Hospital, Linköping, Sweden; 5 Department of Medical Specialists, Institute of Medicine, Motala Hospital, Motala, Sweden; 6 Division of Neurology, Department of Clinical Neuroscience, Karolinska Institute and Karolinska University Hospital, Stockholm, Sweden; 7 Department of Medical Sciences, Uppsala University, Uppsala, Sweden; Uppsala Universitet, SWEDEN

## Abstract

**Background:**

Neurological manifestations in patients with COVID-19 have been reported previously as outcomes of the infection.

The purpose of current study was to investigate the occurrence of neurological signs and symptoms in COVID-19 patients, in the county of Östergötland in southeastern Sweden.

**Methods:**

This is a retrospective, observational cohort study. Data were collected between March 2020 and June 2020. Information was extracted from medical records by a trained research assistant and physician and all data were validated by a senior neurologist.

**Results:**

Seventy-four percent of patients developed at least one neurological symptom during the acute phase of the infection. Headache (43%) was the most common neurological symptom, followed by anosmia and/or ageusia (33%), confusion (28%), hallucinations (17%), dizziness (16%), sleep disorders in terms of insomnia and OSAS (Obstructive Sleep Apnea) (9%), myopathy and neuropathy (8%) and numbness and tingling (5%). Patients treated in the ICU had a higher male presentation (73%). Several risk factors in terms of co-morbidities, were identified. Hypertension (54.5%), depression and anxiety (51%), sleep disorders in terms of insomnia and OSAS (30%), cardiovascular morbidity (28%), autoimmune diseases (25%), chronic lung diseases (24%) and diabetes mellitus type 2 (23%) founded as possible risk factors.

**Conclusion:**

Neurological symptoms were found in the vast majority (74%) of the patients. Accordingly, attention to neurological, mental and sleep disturbances is warranted with involvement of neurological expertise, in order to avoid further complications and long-term neurological effect of COVID-19. Furthermore, risk factors for more severe COVID-19, in terms of possible co-morbidities that identified in this study should get appropriate attention to optimizing treatment strategies in COVID-19 patients.

## Introduction

Coronaviruses are a large family of viruses causing disease in many species including humans. The first identified severe disease in humans caused by coronaviruses was Severe Acute Respiratory syndrome (SARS) emerging in 2002 with a 9.5% mortality rate, followed by Middle East Respiratory Syndrome [1–3] emerging in 2012 with a 34.4% mortality rate [4, 5]. The latest severe disease caused by coronaviruses reported first in Wuhan, China in December 2019 caused severe acute respiratory disease COVID-19 [6]. The most common symptoms of SARS-CoV-2 infection are fever, cough, fatigue, shortness of breath, and muscle soreness [7]. Rhinorrhea, chest tightness, sore throat, nausea, vomiting, diarrhea, headache, ageusia, and anosmia have also commonly been reported among infected persons [8]. While some patients only experience a mild fever, mild fatigue, or even no symptoms [9, 10], severe illness with acute respiratory distress syndrome, respiratory failure, multiple organ failure and death has occurred in other patients [11].

Previous studies addressed the association of obesity and age with COVID-19 severity and poor prognosis [12, 13]. Underlying comorbidities such as cardiovascular disease, hypertension, diabetes, and renal disease are also associated with detrimental outcomes [14, 15]. In addition, neurological manifestations related to COVID-19 have been reported. Fatigue, myalgia, anosmia, ageusia and headache are common neurologic symptoms observed in COVID-19 patients. Dizziness, acute confusion/delirium, agitation, stroke, hypoxic ischemic injury, seizures, coma, and encephalitis have also been reported [16–19].

Here we present a retrospective, observational cohort on data collected from March -June 2020 at two medical centers in Östergötland County, Sweden including 196 consecutive patients with laboratory confirmed diagnosis of COVID-19.

## Materials and methods

### Study population

**Fig 1:**
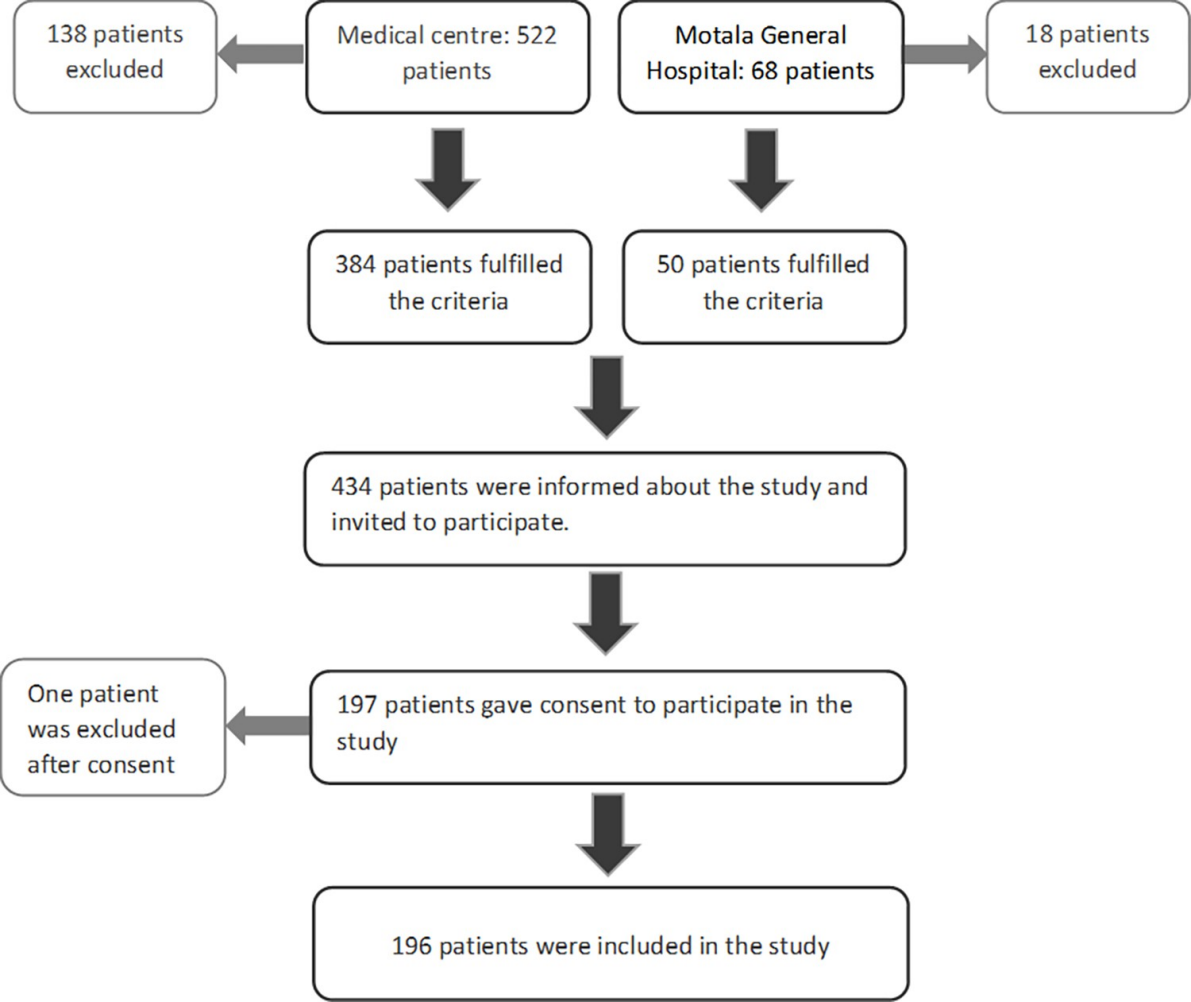


A total of 690 patients were diagnosed with COVID-19 during March 2020 to June 2020 in Östergötland county, including 68 patients at Motala Hospital, and 522 patients from other parts of the county. All adult patients (>18 years) with COVID-19 regardless of if they were treated as outpatients or inpatients with COVID-19 as a primary diagnosis or co-diagnosis, were retrospectively identified through a local diagnosis registry. Patients with negative PCR, deceased patients, presence of protected personal data or a diagnosis of frontal lobe dementia were excluded which resulted in 434 patients eligible for inclusion in the study. We sent a letter to 434 COVID-19 patients (384 patients from Medicine center and 50 patients from Motala Hospital) with information about the aims of study and study procedure and all patients provided informed written consent to have data from their medical records in research. We received 197 consent letters (signed the informed consent) and 196 patients were included in the current study after fulfilling inclusion criteria (Fig 1). All data were fully anonymized and all data handled in according to Data Protection ACT (Personal Data Act, 1998:204).

Demographic and clinical data including neurological symptoms of each study patients were extracted from electronic records by a trained research assistant and physician and collected data were checked by a senior neurologist through medical chart review. Neurological symptoms and signs were categorized as attributed to the central nervous system, peripheral nervous system, or musculoskeletal system.

### Statistics

Mean and standard deviation were calculated for continuous variables (e.g., age) and were summarized using descriptive statistics. Categorical variables were summarized as absolute frequencies and percentages. The software used for statistical analysis was Stata v17.1, StataCorp LLC, College Station, USA.

### Ethics statement

The Linköping Medical Ethics Committee gave their approval to the study (Dnr 2020–02591). The study was conducted in agreement with the declaration of Helsinki (64^th^ WMA General Assembly, Fortaleza, Brazil, October 2013).

## Results

### Baseline demographics

A total number of 196 patients (105 /91 male vs female) with COVID-19 were enrolled during the study. The age of patients ranged from 25 to 92 years, with an average age of 62 years. Eighty-six patients out of 196 were over 65 years old. This population was predominantly Nordic European and the average BMI was 29,5. Ongoing or history of past smoking was reported in 57%, ongoing /past use of oral tobacco (snuff) was detected in 40% and 23% had a history of chronic alcohol overconsumption (Table 1).

**Table 1:** Characteristics of the study patients.

	n	Total known	%
Age, mean (range)	62 (25–92)	196	
≥65 years old	86 (43/43)	196	44%
Sex (male/female)	105/91	196	54%/46%
BMI, mean	29,5	127	
Ongoing/history of smoking	87	152	57%
Ongoing/history of snuffing	21	52	40%
Ongoing /history of alcohol overconsumption	13	56	23%
**Comorbidities**			
Hypertension	97	178	54.5%
Depression and anxiety	81	160	51%
Sleep disorders (insomnia and/or OSAS)	50	165	30%
Cardio- or cerebrovascular disease	46	166	28%
Autoimmune disease	39	156	25%
Chronic Lung disease (Asthma and/or COPD)	38 (37)	159	24% (23%)
Diabetes Mellitus type II	38	164	23%
Allergic Rhinitis/Pollen allergy	27	171	16%
Chronic kidney disease	17	162	10.5%
Thromboembolic disease	14	155	9%
Other psychiatric disorders	5	156	3%

Table 1 shows the overall demographics and comorbidities of study patients. “n” shows the number of patients who replied positive to the corresponding variable, “total known” represents the total number of patients who replied to the corresponding question and the column to the most right shows the percentage of occurrence of each variable in regard to the respective total known cases.

OSAS = Obstructive Sleep Apnea.

COPD = Chronic Obstructive Pulmonary Disease.

The most common pre-existing and concurrent comorbidities found in the medical files were hypertension, depression and anxiety, sleep disorders in terms of insomnia and OSAS, cardio- or cerebrovascular disease, autoimmune diseases (S1 Table), chronic lung diseases mainly in form of Asthma and/or Chronic Obstructive Pulmonary Disease (COPD), diabetes mellitus type 2 and Allergic Rhinitis/Pollen allergy (Table 1).

### Clinical COVID-19 related signs, symptoms and outcome

All COVID-19 related clinical manifestations are listed in Table 2.

**Table 2:** Overall symptoms under first and/or second medical care seek.

Variable	n	Total known	%
Hospitalization	175	196	89%
Average of medical care period (days)	16		
Average of days in Intensive Care Unit (days)	18		
**General symptoms**			
Fever	177	184	96%
Cough	167	184	91%
Oxygen supply requirement	108	134	80%
Dyspnea/shortness of breath	128	179	71.5%
Gastrointestinal symptoms (nausea, vomiting or diarrhea)	94	181	52%
Bacterial or fungal infections	59	160	37%
Respiratory care	37	134	28%
Coagulation disorder	17	188	9%

Table 2 shows the symptoms during first and/or second medical care seek. “n” shows the number of patients who replied positive to the corresponding variable, “total known” represents the total number of patients who replied to the corresponding question and the column to the most right shows the percentage of occurrence of each variable in regard to the respective total known cases.

The most common symptom was fever, followed by cough, need of oxygen supply, dyspnea, gastrointestinal symptoms and bacterial or fungal infections. Among 196 patients, 175 (89%) needed hospitalization during the COVID-19 infection with an average medical care period of 16 days and a mean time-period of 18 days in the Intensive Care Unit (ICU) (Table 2).

Among ICU patients, 33 (73%) versus 12 (27%) were male respective female with an average age of 62 (S2 Table). 82% of ICU patients were intubated. Cardiovascular disease as comorbidity were seen more often in ICU patients compared to non-ICU patients (p<0.005). Otherwise, all demographic variables, including the frequency and type of co-morbidities, were comparable in the patients admitted to the ICU and the non-ICU patients. The duration of medical care at ICU had not any impact on disease severity.

### Neurological manifestations

A total of 145 out of 196 patients (74%) developed at least one neurological symptom during hospitalization for COVID-19 (Table 3).

**Table 3:** Neurological manifestations.

Patients with neurological manifestations during COVID-19	n = 145 (%)	% /196
Headache	63 (43%)	32%
Anosmia and/or ageusia	48 (33%)	23%
Confusion	41 (28%)	21%
Hallucination	25 (17%)	13%
Dizziness	23 (16%)	12%
Sleep disorders (insomnia & OSAS)	13 (9%)	7%
Myopathy and neuropathy	12 (8%)	6%
Numbness and tingling	8 (5%)	4%

Table 3 shows different neurological manifestations among study population. The middle column shows the number of positive cases in regard to each variable and the percentage among total number of patients with neurological manifestation (n = 145). The column to the right shows the percentage among the total number of study patients (n = 196).

OSAS = Obstructive Sleep Apnea.

The most common neurological symptom was headache followed by anosmia and/or ageusia, confusion, hallucinations, dizziness, and disturbance of sleep in terms of insomnia and OSAS. Twelve patients (8%) experienced myopathy and/or neuropathy (Table 3), including three with clinical and electrophysiological verified critical illness polyneuropathy and myopathy among the ICU patients.

The presence of neurological symptoms overall and/or a specific neurological symptom did not correlate with COVID-19 severity with regard to duration of hospital stay and/or need of ICU care. Furthermore, none of the co-morbidities were correlated with occurrence of a specific neurological symptom or presence of neurological symptoms overall.

## Discussion

This retrospective study on 196 symptomatic COVID-19 patients, revealed 145 patients (74%) with at least one neurological symptom such as headache, anosmia and/or ageusia. More severe neurological and mental complications such as confusion and hallucination were almost exclusively observed in mid-older patients (average age of 67).

Many other studies have also reported similar neurological symptoms of which the incidence of overall neurological manifestations has varied broadly from 36% [20] to 73% [21]. Another recent study reported a yet higher frequency, of 84% of neurological symptoms in COVID-19 infected individuals compared to the present study [22]. The high prevalence of neurological manifestations associated with COVID-19 is suggested to be either indirect consequence of thrombotic complication, inflammation, hypoxia, dysregulation of blood pressure, or a direct cause of neurotropic properties of the virus itself [23, 24]. However, more research needs to be performed to understand the pathogenic mechanisms behind neurological disorders.

Similar to our study, the most common neurological symptoms previously reported have been headache, anosmia and/or ageusia [25, 26]. In some studies, with severe cases, stroke, seizure, and encephalopathy has also been reported as neurological symptoms [27]. Moreover, although confusion and hallucination has been considered as atypical and unusual presentation of COVID-19 [28], many independent studies have described these symptoms as a recurrent neurologic manifestation of COVID-19 [29, 30]. In our study, 28% of the patients with neurological and mental manifestations showed confusion and 17% of patients experienced hallucination.

It is also acknowledged that certain underlying host factors and medical conditions pose a greater risk for a more severe COVID 19 disease course. Males have been reported to have a higher likelihood for more severe outcome of COVID-19 [31], which is in line with our study, showing that 73% of ICU patients were males (S2 Table). Previous studies have identified overweight and obesity as risk factors for COVID-19 associated hospitalization and death [32, 33], the risk for hospitalization and ICU admission was lowest among healthy BMIs (18.5–24.9 kg/m^2^) but dramatically increased with overweight (25–29.9 kg/m^2^) and obesity [33]. Interestingly and in line with previous studies, the mean BMI of the current COVID-19 cohort was 29,5 (Table 1). Several studies [25, 34–36] have reported hypertension as the most common comorbidity among COVID-19 patients and as a lead risk factor for developing severe COVID-19 [37], which has been suggested to be associated with upregulation of *ACE2* gene expression, encoding for the receptor used by SARS-CoV-2 [38, 39]. Here, our study also highlighted a broad spectrum of comorbidities in the COVID-19 patients, of which the most common comorbidity was hypertension (54.5%). Interestingly, the second most common comorbidity in the present COVID-19 patients was depression and anxiety (51%, Table 1). This is in line with several other studies highlighting the impact of pre-existing mental disorders and increased probability of developing COVID-19 [40–42].

We also observed a high incidence (30%) of sleep disturbance in terms of insomnia and OSAS among the preceding and concurrent comorbidities in the study cohort, which may indicate that such symptoms are a risk factor to consider (Table 1). The frequency of insomnia in our material is not different from the expected prevalence, but OSAS most certainly exceeds than what is expected. Interestingly, in line with our study, a previous American study covering 5400 COVID-19 infected patients found that people with a pre-existing sleep disorders have more severe outcomes from COVID-19 [43]. However, another study conducted by Goldstein *et al*, on 572 adult patients hospitalized for COVID-19, reported no significant contribution of sleep disorders to outcomes of the illness [44].

In addition to cardio- and cerebrovascular disease and diabetes mellitus type 2, our data also suggests that autoimmune diseases may have some impact on the risk for COVID-19 (Table 1 and S1 Table). An interesting background in this respect are studies of MS, with differences in the outcome related to the choice of immunomodulating drugs [45, 46]. In our material, however, the presence of autoimmune disease and immunoactive therapy made no difference regarding the outcome of the infection, taken from comparison between severe versus non severe COVID-19 disease (ICU versus non- ICU patients). An impact by autoimmune disease has previously been reported [47], which has been suggested to be associated with glucocorticoid administration.

In addition, a study from the Queen Mary University in London [48] suggested that persons with a prior allergic disease, particularly asthma have a lower risk to develop severe COVID-19, perhaps because the expression of *ACE2* is lower in people with allergic asthma and allergic sensitization [39]. However, in our study, asthma, and allergic rhinitis stand up as a comorbidity with relatively high incidences (23% respective 16%) (Table 1). Earlier data has also suggested a higher rate of asthma in patients hospitalized for severe COVID-19 illness [49], although this data as well as our study does not specify whether the asthma is allergic. There are also other studies [50, 51] reporting none significant impact of respiratory allergy on severity of COVID-19.

The current study has several limitations. This is a regional study conducted in a relatively homogeneous cohort. This per se, together with a small number of patients limit our understanding of the true extensiveness of neurological problems associated with COVID-19. Additionally, the study cohort was collected during the very early pandemic when treatment regimen and knowledge of COVID-19 was limited which might have rendered this population particularly vulnerable for a more severe disease course. Despite limitations, our findings are quite well consistent and in line with previous reported neurological manifestations and comorbidities by other groups, and supports the conclusion that neurological manifestations are an important cause of COVID-19, and even suggested to be a primary feature of COVID-19 disease [52] and possibly a risk factor for mortality [53], despite the fact that mechanism behind neurological symptoms are still unknown, which needs to be further elucidated.

## Conclusion

Neurological symptoms were found in 74% of the COVID-19 patients. Several risk factors in terms of co-morbidities, i.e., preceding, and concurrent diseases, were identified which gives an interesting background for further studies on the topic in order to optimizing care for COVID 19-patients in the future including identifying risk groups that should get appropriate attention. This study is planned for a long-term follow up, giving further opportunity to determine the impact of such risk factors in the long-term outcome as well as the odds for post COVID syndrome.

## Supporting information

S1 Table S1 Table shows the characteristics of patients with autoimmune disease, the disease and the treatment where applicable. (DOCX)

S2 Table S2 Table shows the characteristics of ICU versus non-ICU patients. (DOCX)
