# Supplementary material for: Inventory study of an early pandemic COVID-19 cohort in South-Eastern Sweden, focusing on neurological manifestations
Source: PLoS One. 2023 Jan 13;18(1):e0280376. doi: 10.1371/journal.pone.0280376 (PMC9838851; doi:10.1371/journal.pone.0280376)
Supplement: S1 Table — (DOCX) [file pone.0280376.s001.docx]

| **Gender** | **Age** | **BMI** | Autoimmune disease | Immunmodulatory/  suppressive Treatment | Respiratory care at ICU |
| --- | --- | --- | --- | --- | --- |
| F | 66 | 24 | Celiac disease |  |  |
| F | 56 | 32 | Multiple Sclerosis | Interferon beta-1a |  |
| M | 77 | 28,4 | Myasthenia gravis |  |  |
| F | 64 | 24 | Autoimmune Thyroiditides |  |  |
| F | 73 | 25 | Chrohn’s disease |  |  |
| M | 62 | 27,8 | Psoriasis |  | X |
| F | 74 | 31,5 | Sjögren’s syndrome |  |  |
| F | 78 | 30 | Polymyalgia rheumatica |  |  |
|  | 92 | missing | Type 1 Diabetes |  | X |
| M | 82 | 24,4 | Polymyalgia rheumatica |  |  |
| M | 80 | 36,8 | Rheumatoid arthritis |  |  |
| F | 80 | 22,1 | Type 1 Diabetes |  |  |
| F | 78 | 37,5 | Polymyalgia rheumatica |  |  |
| M | 78 | 29,2 | Psoriasis & psoriasisartrit |  |  |
| M | 75 | 31,6 | Rheumatoid arthritis |  |  |
| F | 74 | 23,3 | Polymyalgia rheumatica |  |  |
| F | 74 | 28,4 | Rheumatoid arthritis |  |  |
| F | 73 | 26,3 | Ulcerative colitis | Mesalazine & Azathioprine | X |
| F | 73 | 31,5 | Psoriasis |  |  |
| F | 70 | 29,5 | Type 1 Diabetes |  |  |
| F | 69 | 33,2 | psoriasisartrit | Methotrexate |  |
| F | 67 | 26,9 | Ulcerative colitis |  |  |
| F | 67 | 30,9 | Ulcerative colitis | Mesalazine |  |
| F | 65 | 36,1 | Autoimmune Thyroiditides |  | X |
| M | 65 | 56 | Chrohn’s disease | Mesalazine |  |
| M | 65 |  | Secondary progressive Multiple Sclerosis |  |  |
| M | 64 |  | Ulcerative colitis | Mesalazine | X |
| F | 63 | 28,4 | Graves’ disease |  |  |
| F | 60 |  | Rheumatoid arthritis | Methotrexate |  |
| F | 60 |  | Autoimmune Thyroiditides |  |  |
| M | 59 |  | Multiple Sclerosis | Rituximab |  |
| M | 54 | 23,9 | Axial spondylartrit | Sulfasalazine & Methotrexate |  |
| M | 53 | 29,4 | Ulcerative colitis, Celiac disease | Colazid |  |
| M | 52 |  | Chrohn’s disease | Adalimumab |  |
| F | 49 | 34,9 | Type 1 Diabetes |  |  |
| M | 48 | 34 | Ulcerative colitis & Autoimmune hepatitis |  |  |
| F | 41 | 24,5 | Rheumatoid arthritis | Methotrexate |  |
| F | 40 | 34,8 | Ulcerative colitis |  |  |
| F | 38 | 22,8 | Autoimmune Thyroiditides & Type 1 Diabetes |  |  |
