# Supplementary material for: Inventory study of an early pandemic COVID-19 cohort in South-Eastern Sweden, focusing on neurological manifestations
Source: PLoS One. 2023 Jan 13;18(1):e0280376. doi: 10.1371/journal.pone.0280376 (PMC9838851; doi:10.1371/journal.pone.0280376)
Supplement: S2 Table — (DOCX) [file pone.0280376.s002.docx]

**Supplementary table 2. Characteristics of the ICU/Non-ICU patients, in the COVID-19 patient cohort**

| **Variable** | **Intensive care, n=45** | **Non-intensive care, n=151** |
| --- | --- | --- |
| Gender | 33/12 (Male/female) | 72/79 (Male/female) |
| Age, mean | 62 | 62 |
| BMI, mean | 29 | 29 |

Supplementary table 2 shows the characteristics of ICU versus non-ICU patients.
